# Supplementary material for: A type III secretion system is required for Bordetella atropi invasion of host cells in vivo
Source: PLoS Pathog. 2026 Feb 13;22(2):e1013949. doi: 10.1371/journal.ppat.1013949 (PMC12923130; doi:10.1371/journal.ppat.1013949)
Supplement: S1 Fig — Bacteria are pseudo-colored in green. Yellow arrowheads indicate apical junctions. White arrowheads indicate basement membrane. lu, lumen, mu, muscle. Scale bars are 500 nm. (DOCX) [file ppat.1013949.s001.docx]

**
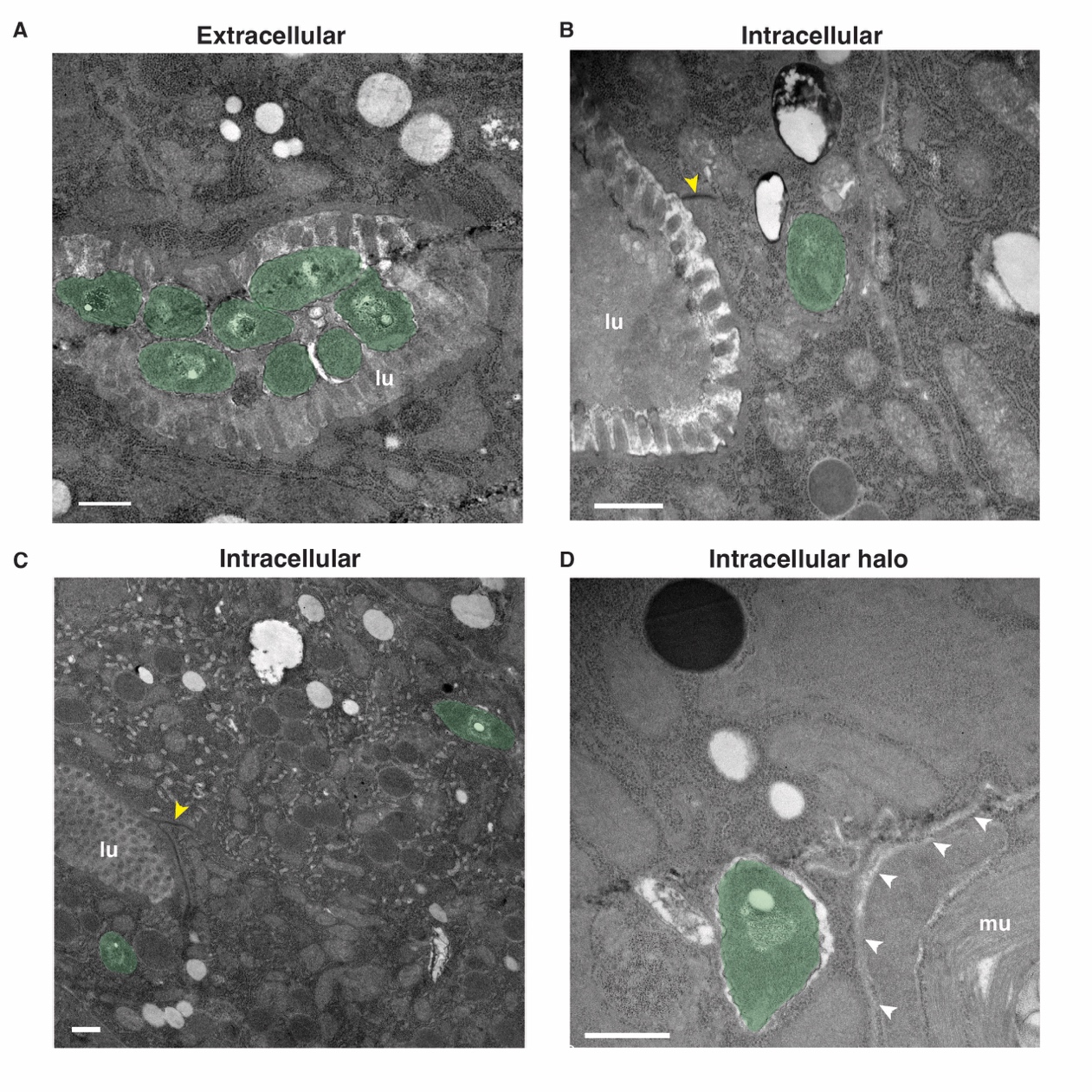
**

S1 Fig. **Different phenotypes of wildtype *B. atropi* at 22 hpi by transmission electrion microscopy**. Bacteria are pseudo-colored in green. Yellow arrowheads indicate apical junctions. White arrowheads indicate basement membrane. lu, lumen, mu, muscle. Scale bars are 500 nm.
