# Supplementary material for: A type III secretion system is required for Bordetella atropi invasion of host cells in vivo
Source: PLoS Pathog. 2026 Feb 13;22(2):e1013949. doi: 10.1371/journal.ppat.1013949 (PMC12923130; doi:10.1371/journal.ppat.1013949)
Supplement: S2 Fig — (A) Percent animals infected when exposed to putative T3SS effector knockout strains. (B) Growth curves of different knockout strains compared to WT. Graphs show means with SD from 2 independent replicates, *, p = 0.0273, ns, non-significant by one-way ANOVA. (DOCX) [file ppat.1013949.s002.docx]

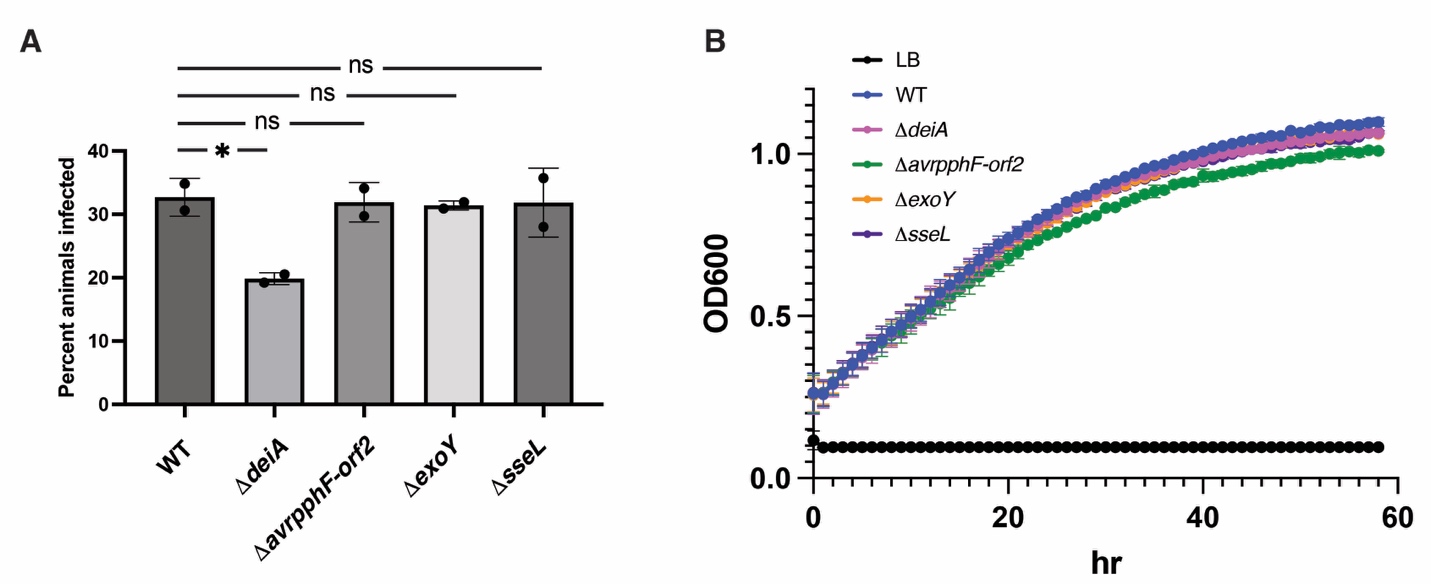


S2 Fig. **Effects of putative T3SS effector knockouts**. **A**. Percent animals infected when exposed to putative T3SS effector knockout strains. **B**. Growth curves of different knockout strains compared to WT. Graphs show means with SD from 2 independent replicates, *, p = 0.0273, ns, non-significant by one-way ANOVA.
