# Supplementary material for: A type III secretion system is required for Bordetella atropi invasion of host cells in vivo
Source: PLoS Pathog. 2026 Feb 13;22(2):e1013949. doi: 10.1371/journal.ppat.1013949 (PMC12923130; doi:10.1371/journal.ppat.1013949)
Supplement: S3 Fig — (A) ΔdeiA was complemented with a WT copy of deiA under control of L-rhamnose inducible promoter. Induction at different concentrations (0.5 or 3 mg/mL) results in growth defects compared to uninduced, the original knockout strain or the WT. Graph shows means with SEM from 6 technical replicates. (B) Number of initial invasion events of induced complemented ΔdeiA compared to uninduced condition and WT at 0.5 mg/mL. Graph shows means with SD from 2 independent replicates for a total of at least 13 infected animals, *, p = 0.0126, ns, non-significant by Kruskal-Wallis test followed by Dunn’s tests. (DOCX) [file ppat.1013949.s003.docx]

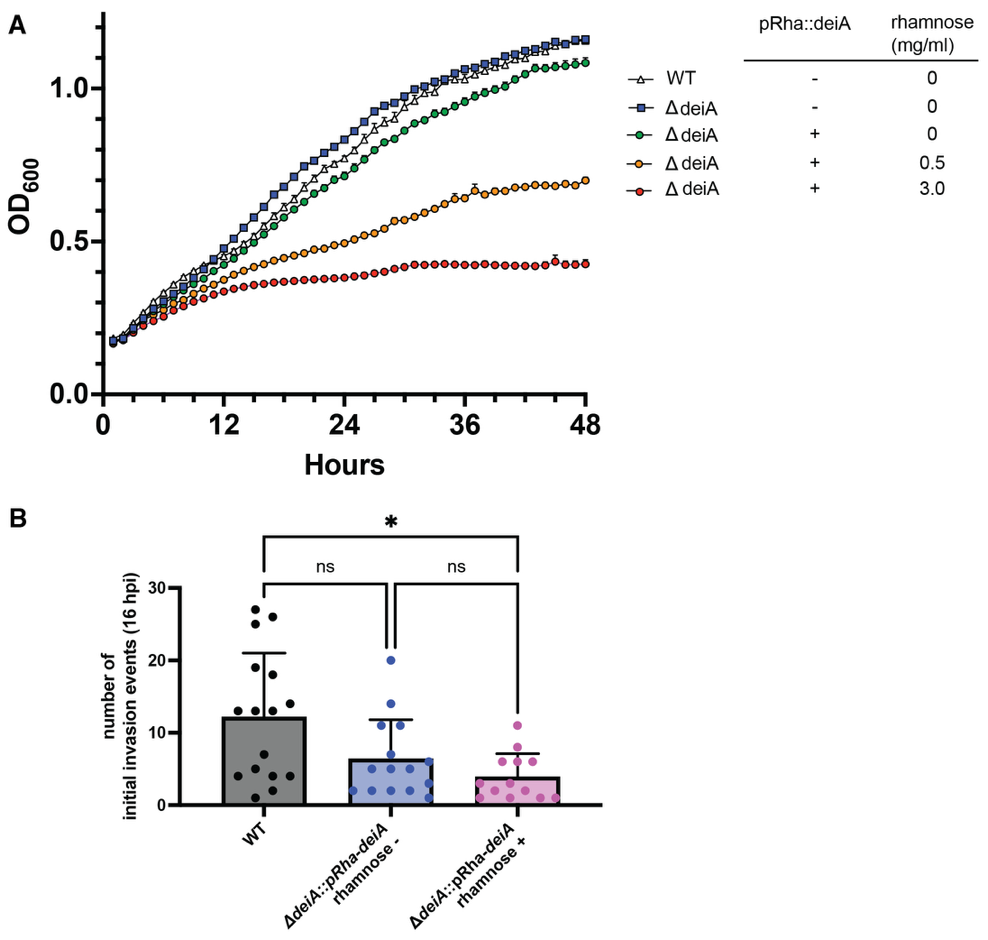


S3 Fig. **Growth defects of overexpressing *deiA* in Δ*deiA* background**. **A**. Δ*deiA* was complemented with a WT copy of *deiA* under control of L-rhamnose inducible promoter. Induction at different concentrations (0.5 or 3 mg/mL) results in growth defects compared to uninduced, the original knockout strain or the WT. Graph shows means with SEM from 6 technical replicates. **B**. Number of initial invasion events of induced complemented Δ*deiA* compared to uninduced condition and WT at 0.5 mg/mL. Graph shows means with SD from 2 independent replicates for a total of at least 13 infected animals, *, p= 0.0126, ns, non-significant by Kruskal-Wallis test followed by Dunn’s tests.
