# Supplementary material for: A type III secretion system is required for Bordetella atropi invasion of host cells in vivo
Source: PLoS Pathog. 2026 Feb 13;22(2):e1013949. doi: 10.1371/journal.ppat.1013949 (PMC12923130; doi:10.1371/journal.ppat.1013949)
Supplement: Attachment — Submitted filename: Tran et al 2025_Responses to reviewers.docx [file ppat.1013949.s008.docx]

We thank the reviewers for their thoughtful analysis of our manuscript. We have addressed all of the concerns below.

**Part II – Major Issues: Key Experiments Required for Acceptance**
Please use this section to detail the key new experiments or modifications of existing experiments that should be absolutely required to validate study conclusions.

Generally, there should be no more than 3 such required experiments or major modifications for a "Major Revision" recommendation. If more than 3 experiments are necessary to validate the study conclusions, then you are encouraged to recommend "Reject".

Reviewer #1: 1) The authors report the identification of DEGs in B. atropi in response to infection (Fig 5A-C) but there does not appear to be a supplemental table showing the DEGs or their fold change/statistical significance. Only a supplemental table of the Proline Rich Motif proteins that change. I think a full table of the DEGs should be added to the manuscript and presumably this should already be data the authors have in hand.

Thank you to the reviewer for pointing out this omission. We have added full tables of DEGs (genes with Log2 Fold Change >2 or <-2 and Padj < 10^-3^). These data have been added as Supplementary Table 1 (line 369). The original Supplementary Table 1 showing the list of proline-rich motif T3SS effector candidates is now Supplementary Table 2 (line 372) in the revised manuscript.

Reviewer #2: (No Response)

**Part III – Minor Issues: Editorial and Data Presentation Modifications**
Please use this section for editorial suggestions as well as relatively minor modifications of existing data that would enhance clarity.

Reviewer #1: 2) Along the same lines as point 1, it is very interesting that T3SS genes are among the most increased in abundance. Are T1SS or T2SS components also among the DEGs? Or T6SS genes? Figure 1 of this manuscript does a good job showing that T6SS’s are dispensable for this infection, but does not also KO the of T1SS or T2SS’s despite reporting that their genome analysis identified one T1SS and 1 T2SS in B. atropi. The logic for not focusing on T1SS or T2SS is that they do not inject effectors into target cells and only secrete proteins into extracellular space. However, I still think it would have been more complete to also KO a core component of the T1SS and T2SS’s given the logic presented and think the results would have been interesting either way (are/are not required). This is especially true if T1SS or T2SS genes were also among the DEGs. However, I do not feel that this is not absolutely required for the claims of this manuscript, simply that it would help with completeness and characterization given ordering of the manuscript leading with one type of SS is not required.

We agree with the reviewer creating knockouts of the T1SS or the T2SS to look for a role in infection could be interesting. However, when we looked at the DEGs for in vivo infection we found that none of the components of these two secretion systems made it to the list, even if we changed the Padj cutoff = 0.05 (larger than our current list) and FoldChange =2 (Log2FC =1).

3) Could the authors provide a deiA rescue experiment in figure 5 analagous or similar to their rescues in Fig. 2? The observation for the deiA mutant bacteria is a core new claim and given that it’s only one mutant I think rescue or a 2nd allele should be easy enough to provide and important

We agree with the reviewer that a rescue experiment would strengthen the conclusion that *deiA* functions as a T3SS effector. To this end, we generated two independent *deiA* complementation constructs: one in which *deiA* is expressed from the same L-rhamnose–inducible promoter (Prha) used for T3SS rescue elsewhere in this manuscript, and a second in which *deiA* is expressed from a constitutive Ptac promoter (pANT4).

However, in both cases, the *deiA* rescue strains exhibited a pronounced growth defect relative to the Δ*deiA* background strain and wild-type controls. We have added Supplementary Figure S3 to demonstrate that rhamnose-induced expression of *deiA* causes a severe in vitro growth defect, suggesting that uncoupling *deiA* expression from the rest of the T3SS machinery is toxic to the bacteria.

As a result, when these rescue strains were tested in vivo, it was not possible to distinguish whether the observed reduction in invasion was due to impaired host interaction or simply a consequence of reduced bacterial growth or fitness. We therefore concluded that these rescue experiments do not allow for a meaningful interpretation of *deiA*’s role in invasion and have not included them as primary evidence.

These results have now been included in the revised manuscript (lines 393-399) and Fig. S3 in the revised manuscript.

4) The authors present data showing that exoY mutant bacteria produces a small by statistically significant effect on invasion. Would an deiA and exoA double mutant be even more defective in invasion? Do the authors think these two potential effectors might function in parallel?

It is difficult to predict the phenotype of a *deiA exoY* double mutant with respect to host invasion without a clearer understanding of how these effectors impact the host cytoskeleton. One possibility is that DeiA and ExoY function within the same pathway, for example if DeiA promotes actin nucleation while ExoY acts downstream to bundle or stabilize filaments. In such a model loss of DeiA alone might already produce a near-maximal defect. Alternatively, DeiA and ExoY could act in parallel, either by independently nucleating actin or by affecting distinct aspects of cytoskeletal remodeling. In this case, a double mutant might be expected to show a stronger invasion defect than either single mutant. Our current data do not allow us to distinguish definitively between these models. Notably, the *exoY* mutant shows a modest but statistically significant invasion defect, whereas the *deiA* mutant exhibits a stronger phenotype, which could argue against a strictly linear pathway but is also consistent with partial functional redundancy or compensation by additional, as-yet-unidentified effectors. The relationship between DeiA and ExoY is an important question for future work on T3SS effectors in *B. atropi*. We have added some of this analysis in the Discussion (lines 465-468) in the revised manuscript.

5) I personally have trouble seeing/identifying villi confidently in some of the EM images provided in Fig 3A, particularly for the sctC mutant. If better images are available or potential better highlighting of what is being measured and how it was chosen I think it would be helpful for interpreting the quantification in Fig. 3B.

To improve Figure 3A, we have highlighted the microvilli that were measured. For these images, we specifically measured microvilli that were in the same plane as the apical membrane, and not microvilli fragments disconnected from cytoplasm. We have updated the Fig. 3A legend (lines 287-288), and main text lines 258-261 in the revised manuscript.

Minor point:
6) E. coli is not italicized in line 104

We fixed this typo.

Reviewer #2: Figure 1 C and D, Figure 2 D, Figure 5 F and G, and Figure S2 A: Data are pooled, how was variation between independent experiments handled? (i.e was a test for batch effects done?). Please indicate in figure legends and/or materials and methods.

For these experiments, we validated that the averages between experimental replicates looked similar to each other. A sentence has been added to the Methods to address this (lines 658-659) in the revised manuscript.

Figure 1 B and D What is the difference between the filamentation phenotype shown in the representative microscopy figures in Figure 1B and the A-P infection length/morphology phenotype shown in Figure 1 D? Does the data in Figure 1D represents a measurement of the length of filaments shown (representatively) in the microscopy images?

The anterior-posterior (A-P) infection length used in Fig. 1D is a proxy measurement that correlates well with filamentation in vivo (see doi: 10.1038/s41467-022-28297-6). It is not a direct measurement of filament length. Instead, the length of each contiguous infection in an animal was measured along the A-P axis and summed. This total infection length was normalized to the A-P length of the animal giving the normalized infection length. We have added clarification in the Methods of the manuscript (lines 649-652) in the revised manuscript.

Figure 2A: I suggest to remove all the labels with abbreviations for the subunits that are not relevant since they are also not explained in the figure legend.

We have removed the abbreviations for the subunits in Fig. 2A as suggested.

Figure 2B It is difficult to see that the bacterial mutants only colonize the lumen of the host intestine and are not intracellular, especially with the wildtype control missing.

We have added the WT control to Fig 2B to better highlight the loss in invasion in the T3SS mutants.

Figure 2D: what is the sample size?

We have added the sample size to Figure 2D as suggested

Figure 4 A: could the arrows also be added to the merged image? It then may be easier for the reader to see the engulfment of the bacteria by host protrusions.

We have made this adjustment to Fig. 4A as suggested.
